# Microplastics in personal care products and cosmetics in Sri Lanka

**DOI:** 10.1016/j.heliyon.2024.e29393

**Published:** 2024-04-16

**Authors:** Sachith Gamage, Yohan Mahagamage

**Affiliations:** Centre for Environmental Studies and Sustainable Development, The Open University of Sri Lanka, Nugegoda 10250, Sri Lanka

**Keywords:** Microbeads, Policies, Baseline study, Regulation

## Abstract

In the Sri Lankan context, the lack of baseline studies to mitigate microplastic emissions through personal care and cosmetic products poses a huge problem. Hence this study serves as the first scientific investigation to analyze and characterize microplastics in selected personal care and cosmetic items available in the Sri Lankan markets. Fifteen brands representing five categories (face wash, facial scrubs, baby creams, shaving creams, and skin creams) of personal care and cosmetic items served as the basis for this investigation. Based on a questionnaire survey, from each category, three highly utilized brands were chosen and triplicates from each brand were used for the analysis. All samples were treated with the Fenton reagent to extract microplastics. Then through Nile red staining suspected microplastic were screened and characterized through FT-IR spectroscopy. The Nile Red analysis revealed seven brands of the fifteen to be stained with Nile Red and demonstrate luminance properties under UV light. However, FT-IR analysis proved only six brands contained actual microplastics. Low-density polyethylene and ethylene-propylene copolymer were the dominant types of microplastic. Most microplastics were irregularly shaped and white in color with sizes ranging from 238.55 ± 50.74 to 450.69 ± 174.9 μm. An emission estimation revealed that products FS-01 and FW-03 contain 3.36 ± 0.20 g and 0.2 ± 0.05 g of isolatable microplastics per product. While the present study provides scientific evidence for the availability of microplastics in products in Sri Lankan markets, it also provides a great opportunity to develop relevant policies and regulations to control them.

## 1 Introduction

Microplastics are plastic particles less than 5 mm in size in their largest dimension [1] and microplastic pollution is becoming a more severe and problematic environmental and social problem around the world. Microbeads, a type of primary microplastics are synthetic polymer particles that are greater than 0.1 mm and lesser or equal to 5 mm in size and can be found in many personal care and cosmetic products [2]. Microbeads are mainly used as an abrasive scrubber, exfoliating agent, a bulking agent, to control the release of active ingredients and to extend the shelf life of many products such as cosmetics and personal care products [3,4]. As most of these products are commonly referred to as down-the-drain products, incorporated microplastics will either enter directly into the natural environment or wastewater treatment facilities. However, traditional wastewater treatment plants cannot remove microbeads from domestic and industrial effluent. During the treatment process, 99 % of the microplastics will be settled with sludge, which will ultimately end up in the terrestrial environment. The remaining 1 % will released into the aquatic bodies through effluent. As studies indicate United States of America alone discharged trillions of microbeads into water bodies each day [3]. Since microbeads are not rapidly degradable in the natural environment this has become a huge environmental problem. To worsen the effects, microbeads can absorb heavy metals and act as vectors for heavy metal contaminations in aquatic environments [5].

As global literature indicates, microbeads or microplastics could be found in a range of consumer goods. As Guerranti et al [6] state personal care products could contain about 50,391 microbeads per 1 g of the product and with every single use 229,000 microbeads could be introduced into the domestic sewage systems. In Macao, China, an evaluation of personal care products found that 70 % of the surveyed products contain microbeads. Primarily the found microbeads were polyethylene and an estimated 37 billion microbeads per year were released into the environment escaping the wastewater treatment plants [7]. Confirming a similar emission quantity (39 tons) Lei et al [8] found that 7.1 % of Facial cleansers available in China contain 25.04 ± 10.69 mg/g microplastics while 2.2 % of shower gel products contain 17.08 ± 7.50 mg/g microplastics respectively. To demonstrate the excessive usage of microplastics in personal care products, Duis and Coors [9] discussed that countries situated in the watershed of the North Sea are estimated to use 2300 t of microplastics in their personal care products per year. They further discussed that assuming 90 % of microplastic removal rate in wastewater treatment plants and discharge of all wastewater from these countries would account for approximately 1 % of total microplastics in the northern sea. Ultimately mass emissions from personal care product-derived microplastics were calculated to be 1.2 × 10^4^ tons per year and it was further calculated that from 1970 to 2019 environment has been exposed to up to 3 × 10^5^ tons of personal care product-based microplastics [10].

Microplastics can enter human bodies through drinking water, contaminated seafood, and air. While the full scale of microplastic-related health problems is largely unknown, it has been experimented to have endocrine-disrupting effects on humans [11,12]. Bioaccumulation and biomagnification of heavy metal and persistent organic pollutants (POPs) through microbeads is the most alarming consequence of microbeads pollution and many aquatic animals mistakenly consume these beads and face life-threatening situations. Considering the adverse effects of microbeads, 14 countries have banned the usage of non-degradable microbeads on products [13].

The lack of local studies related to microplastics in personal care products and cosmetics poses a threat to public health and the environment. Furthermore, the absence of enforcement mechanisms and the specific regulations for microbeads in Sri Lanka, allows microplastics to persist in these products. The present study results therefore, could be used to develop legal frameworks for the least studied and crucial environmental problems such as microplastic pollution in the country.

## 2 Methodology

### 2.1 Sampling

Personal care products and cosmetics related to the following five categories were used for the selection of product samples.

i. Facial scrubs
ii. Skin creams
iii. Shaving creams
iv. Face wash
v. Baby products

Three different brands from each category were then selected (A total of 15 brands) and triplicates from each brand were taken out of different production batches (Table 1). Each category contained natural, synthetic, male, female, and baby products as follows. Ayurvedic or products with natural raw materials were categorized here as natural products while products containing mostly chemicals were categorized as synthetic products. After selecting each product, their country of origin, ingredient list, and other relevant information were recorded (Table 1).

**Table 1:** Details of collected samples.

Product category	Brand ID	Country of Manufacturing	Ingredients list	The weight/volume of the product	Sampling amount
Skin creams	SK-01	Pakistan	Unavailable	40 g	1 g
	SK-02	India	Available	50 g	1 g
	SK-03	India	Available	50 g	1 g
Shaving cream	SH-01	Sri Lanka	Available	100 mL	1 g
	SH-02	Sri Lanka	Available	60 mL	1 g
	SH-03	India	Available	50 g	1 g
Baby products	BP-01	Sri Lanka	Available	100 mL	1 g
	BP-02	Sri Lanka	Available	200 mL	1 g
	BP-03	India	Available	100 mL	1 g
Face wash	FW-01	India	Available	50 mL	1 g
	FW-02	India	Available	50 mL	1 g
	FW-03	Sri Lanka	Available	50 mL	1 g
Facial scrubs	FS-01	Sri Lanka	Available	120 mL	1 g
	FS-02	Sri Lanka	Available	120 mL	1 g
	FS-03	Sri Lanka	Available	50 mL	1 g

### 2.2 Extraction of microplastics

Standard microplastic extraction methods were performed and modified to an extent to isolate microbeads/microplastics from personal care and cosmetic products [[14], [15], [16], [17]]. 1 g of each product was weighed and transferred into a 100 ml beaker. Then, 2 ml of 30 % H_2_O_2_ and 1 ml of catalyst (Fenton reagent) were added [18]. The samples were allowed to react at room temperature for 10–15 min. Once the reaction was completed 100 ml of distilled water (45 °C) was added to the sample and stirred vigorously with a glass rod. Then, the samples were first filtered through a 3 mm mesh net. By this first filtration larger particles, especially those that were available in the natural products were removed. Further, as many products were soapy it was proven difficult only to use the membrane filter papers to filter the solutions. By this initial filtration, this problem was evaded up to some extent. Next, the filtrate was vacuum filtered through 0.45 μm membrane filter papers (Whatman). and the filter papers were oven-dried (Lab Kits - drying oven) at 37 °C for 14 h. Similarly, microplastics in 1 mL of a product sample were calculated through gravimetric analysis. This was useful in estimating the microplastic emission from the usage of one product. Most importantly, to ensure the accuracy of the results, several strategies were followed. Primarily, microplastic-free distilled water was used to top up the digested solutions. To obtain microplastic-free water distilled water was filtered using a 0.45 μm membrane filter paper and stored in glass bottles for future use. Moreover, the used filter papers were first randomly checked for the availability of microplastics using Nile Red staining. Once the absence of microplastics in the filter papers was ensured the filtration procedure was carried out. Further, the extracted microplastics were kept in the filter paper, and using aluminum foil they were packed and labeled accordingly. No plastic equipment was used during any part of the extraction process and these quality assurance steps were followed for all the samples.

### 2.3 Nile red analysis

Nile Red is a lipid-soluble fluorescent dye that has been mostly used to evaluate the lipid content of animal cells and microorganisms and it is solvatochromic. Therefore, its fluorescence emission spectrum changes with the polarity of the environment in which Nile Red is present [19]. Such features of Nile Red dye provide a possibility to identify microplastics based on their hydrophobicity. However, it is important to note that the interaction of Nile Red and the microplastics depends on the chemical characteristics of the type of plastic [20]. In many samples, plastic-like material could be easily misidentified as microplastics. Thus, Nile Red analysis is a useful method to easily distinguish microplastics among complex matrices. As a result, Nile red staining was carried out on extracted samples to identify products that may contain microplastics. Nile red (Sigma-Aldrich) was prepared at a concentration of 8 μg/ml [21] and filter papers were stained immediately with a few drops of the solution. Stained filter papers were observed under a stereomicroscope by applying an ultraviolet wavelength of 365 nm using UV light [22]. Then, samples were further analyzed to characterize the material present.

### 2.4 Characterization of microplastics

**Fig. 1:**
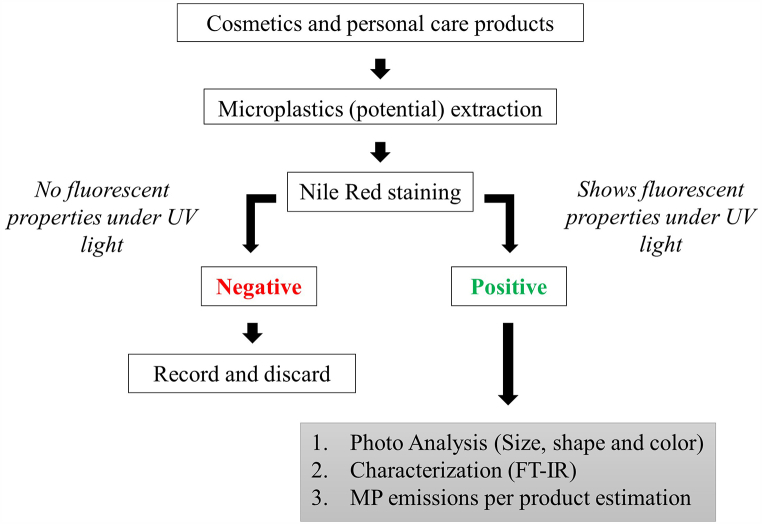
Flowchart of microplastic identification.

Characterization of the potential microplastics in the products was important to confirm their composition and beneficial to understanding the physical and morphological characteristics. Further, quantification of microplastics was carried out to estimate the microplastic emission per product usage. A stereomicroscope (Inskam315-W wifi digital microscope) was used to take images of the microbeads and microplastic presence in the samples. Then images were analyzed using Image J software to understand the size, shape, and color of the microplastics [23]. FT-IR (Fourier Transform Infrared) spectroscopy (Bruker – Alpha) was carried out to identify the composition of extracted microplastics. The Attenuated Total Reflection (ATR) method was used to characterize the potential microplastics and used an open-source spectrum library to identify the polymer type [24]. To increase the accuracy of the analysis at least 03 particles from each brand were used. However, in some products, some particles were hard to isolate. In that case, the extract was carefully scraped off the filter paper and checked for composition. Fig. 1 illustrates the process applied in this study for microplastic identification.

## 3 Results and discussion

In all the product brands except for SK-01, the product packaging included an ingredient list. As this is a requirement for consumers to make educated buying decisions, there should be a proper mechanism to enforce it. The product FW-03 has stated polyethylene is used as an ingredient, thus microbeads have been added into FW-03 intentionally. But none of the other products has mentioned any type of synthetic polymers in their ingredients.

### 3.1 Nile red and FTIR analysis

**Fig. 2:**
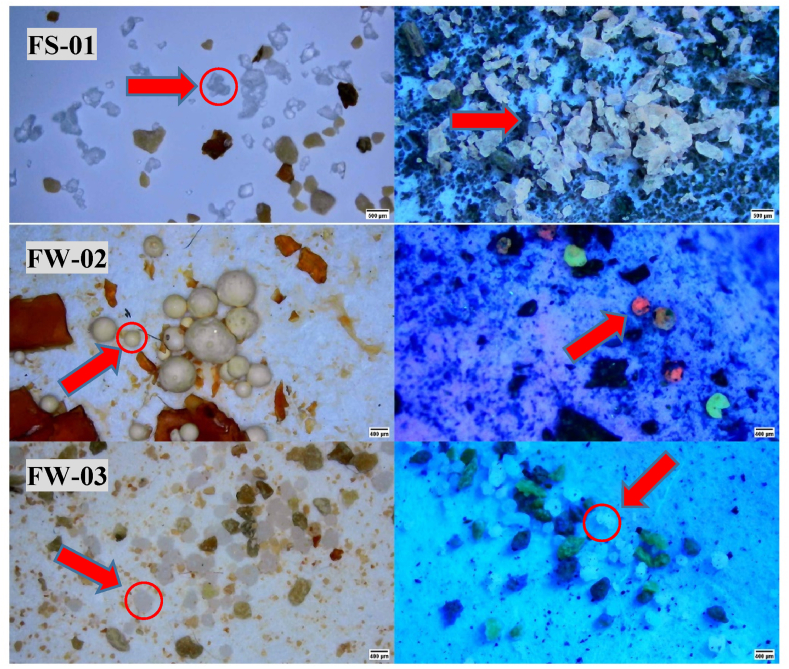
Suspected microplastic particles (left) and Nile red stained particles under ultraviolet light (right). (For interpretation of the references to color in this figure legend, the reader is referred to the Web version of this article.)

Out of 15 brands, 7 brands were positive for Nile red analysis Those were FW-03, BP-02, SK-01, BP-03, FW-02, FS-02, and FS-01 (Fig. 2). Their morphological characteristics were then identified using Image J software. As summarized in Table 2, irregularly shaped or round-shaped MP's were mostly found in whitish or transparent colors. In many literature sources, similar findings have been reported all over the world. Suardy, Tahrim & Ramli [25] explained similar findings in Malaysia. They found MP's from PCPs in irregular and spherical shapes with white or colorless properties. In Macao, China; locally available PCP's found to contain irregularly shaped fragments and spherical microbeads of white or white transparent colors while the same results have been found in some exfoliating products in Spain [7,26]. As the literature suggests most microplastics found in personal care products are irregularly shaped rather than spherical. The main reason for this is, irregularly shaped microplastics have more surface area and greater friction. Which is ideal for the specific functions it was used [27]. Table 2, further illustrates the relationship between the availability of microplastics with their origins. In this case, both locally manufactured and imported products were found to be sources of microplastics. This scenario demonstrates the need for efficient and functioning policies and regulations to manage both the manufacturing process and the importation of cosmetic and personal care products.

**Table 2:** Summary of the suspected microplastics found in the products.

Sample	Shape	Color	Polymer type	Size (mean ± SD μm)	Emission (g/product)	Source origin
SK-01	Irregular	Whitish	Ethylene propylene	320.22 ± 124.60	–	Pakistan
BP-02	Irregular	White	Low-density polyethylene	314.93 ± 98.34	–	Sri Lanka
BP-03	Round	White	Ethylene propylene	238.55 ± 50.74	–	India
FW-03	Round	White	Low-density polyethylene	301.38 ± 83.71 -	0.2 ± 0.05	Sri Lanka
FS-01	Irregular	Transparent	Low-density polyethylene	450.69 ± 174.9	3.36 ± 0.20	Sri Lanka
FS-02	Irregular	White	Polyethylene	280.78 ± 52.11	–	Sri Lanka

*Sample SK-02, SK-03, SH-01, SH-02, SH-03, BP-01, FW-01, FS-03 did not have any MPs present. Sample FW-02 contained Ethyl cellulose microbeads.

**Fig. 3:**
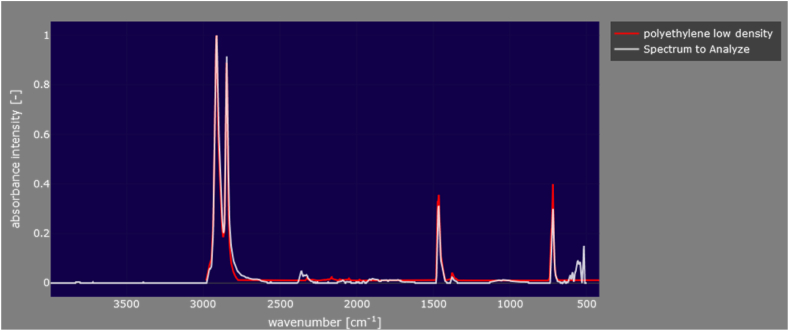
FT-IR spectrum of FW-03 (white-colored spectrum) and Similarity of FW-03 spectra to LDPE spectra (Red colored spectrum). (For interpretation of the references to color in this figure legend, the reader is referred to the Web version of this article.)

Nile red positive samples were further analyzed for FT-IR to understand the composition of suspected particles. For each suspected particle FT-IR spectrums were taken in ATR mode. While they were first identified in an example spectrum library for material characterization, the Open Spacy spectrum library was used to confirm the identification (Fig. 3).

**Fig. 4:**
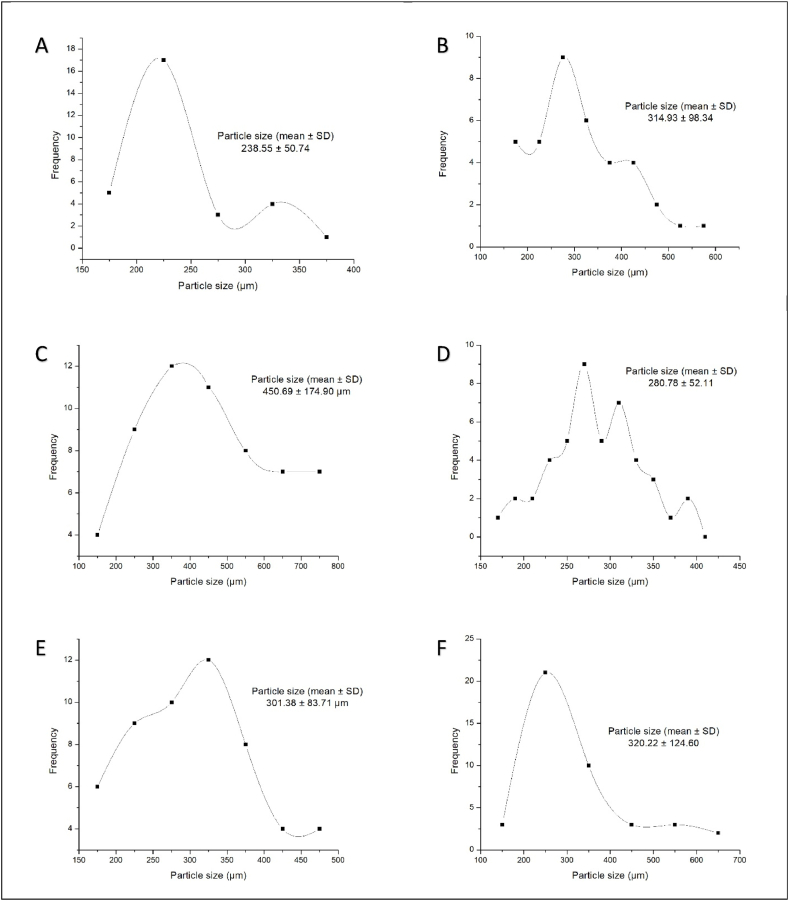
Microplastic size distribution curves for A – *BP-03*, B– *BP-02*, C – *FS-01*, D – *FS-02*, E − *FW-03* and F – *SK-01*.

Accordingly, Table 2 summarizes the characterization of each suspected particle. Low-density Polyethylene, Polyethylene, and ethylene-propylene were the common polymers among the found microplastics. However, one product (FW-02) contained ethylcellulose microbeads which were found to be biodegradable, hydrophobic cellulose [28,29]. Based on the photo analysis size distribution curves were developed for BP-02, BP-03, FS-01, FS-02, FW-03, and SK-01 (Fig. 4). Accordingly, the highest mean particle size was found in FS-01, and the lowest in BP-03 (Fig. 4)

### 3.2 Amount of microplastics per sample/product

As Gamage and Mahagamage [30] discussed, in a sample population, skin creams were used by 62 %, face wash was used by 63 %, and facial scrubs were used by 39 %. Baby care products were used by almost all of the parents. In this scenario, around 94 % of the respondents removed the applied products from their bodies by bathing and washing. Ultimately the generated wastewater entered into gullies (69 %), municipal wastewater collection systems (25 %), or natural water bodies (6 %). Therefore, it is clear that if the products were to contain microplastics, they would ultimately end up in natural water bodies. In this case, estimating the microplastic emissions from the PCPs and the cosmetics is an essential step for minimizing pollution and encouraging policy development activities. To get a sense of microplastic emissions from the studied products, microplastics per product were analyzed for products containing isolatable microplastics.

**Fig. 5:**
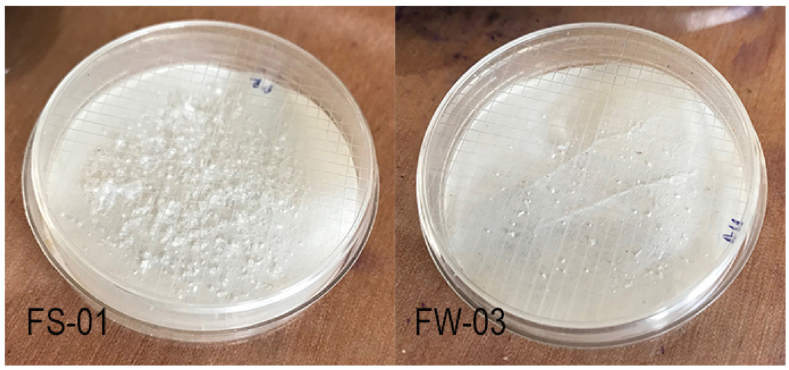
Density-separated microplastics from FS-01 and FW-03 (From 5 ml of sample).

The estimations were specifically done for two specific brands (FS-01 and FW-03). The main reason for this limitation was based on the density separation of microplastics from the tested products. While microplastics from FS-01 and FW-03 were easily density separated (Fig. 5), the remaining brands were proven hard to density separate using the available facilities. Since this could lead to false results in the gravimetric estimation process (due to non-microplastic particles) estimations were not carried out for the other 4 brands. However, since these estimations are important to understand the severity of the microplastic emissions, further studies should be conducted using novel technologies and improved procedures.

When considering the emission (estimated) of low-density polyethylene FS-01 emits 3.36 ± 0.20 g of microplastics, and FW-03 emits 0.2 ± 0.05 g of microplastics per product. While per-product emissions are not found in scientific studies to compare with the present findings, microplastic emissions from PCPs and cosmetics are calculated according to other factors by many scientists. Praveenta et al [31] estimated that Malaysia alone discharges 0.199 trillion microplastics annually from PCPs and cosmetics. In Slovenia, an average amount of 15.2 mg of microplastics was estimated to be released into the environment by a person, making 112.5 million microplastic emissions per day [32]. The United States of America is estimated to be releasing 8 trillion microbeads into the aquatic environments per day [3]. Comparison with the available data suggests the need for a universally accepted method to calculate the microplastic emissions from PCPs and cosmetics. In that way, a clear understating of the present problem could be understood globally rather than locally.

The present study found low-density polyethylene in FW-03, BP-02, and FS-01 samples. FS-01 is the only product that has mentioned the incorporation of LDPE in the product ingredient list. The main reason for using LDPE microplastics in FS-01 is to facilitate the exfoliating nature. While we found microplastics in BP-02, the role of low-density polyethylene in BP-02 is not clear. Low-density polyethylene (LDPE) is one of the most commonly used polymers around the world with an annual production rate of 19 Mt [33], even though the production of low-density polyethylene is causing more environmental pollution than the other polymers [34]. It has good strength, flexibility, low cost and most importantly it is chemically inert [35]. This is one of the main reasons for using low-density polyethylene in cosmetics and personal care products as exfoliating agents. In Malaysia, LDPE microplastics have been observed in several facial scrub and facial cleansing products similar to the present study [31]. Moreover, LDPE microplastics have been observed in numerous face scrub products in China, Grahamstown; South Africa, and many more countries [15,36].

In number of studies, many scientists have found LDPE microplastics contaminating oceans, food sources, and more. This data demonstrates the danger of this microplastic compound. As Wang et al [37] discussed the presence of LDPE microplastics significantly alters the temporal turnover of soil microbial communities. Therefore, the productivity of the soil gets lower drastically over time. Similarly, Rong et al. [38] observed the presence of LDPE microplastics in soil tends to impact soil bacterial network structure and alter the functional groups involved in the carbon and nitrogen cycle. LDPE microplastics were found to be suppressing the well-being of *Acropora formosa* [39]. Further, these particles caused significant bleaching and necrosis to *Acropora formosa,* and the presence of such microplastics was found to be causing direct and indirect impacts on coral polyps by inhibiting photosynthesis.

In this study, SK-01 and BP-03 were found to contain ethylene-propylene copolymer. Ethylene-propylene copolymer (CAS number 9010-79-1) is mostly used in cosmetics and personal care products as an abrasive agent, bulking agent, and film-forming agent [40]. While the ingredient could be officially found in the International Nomenclature Cosmetic Ingredients (INCI) inventory, the present study couldn't find any evidence in available literature related to finding ethylene-propylene copolymer in PCPs and cosmetics as microplastic contaminants. Nevertheless, similar to LDPE microplastics, ethylene-propylene copolymers are found to be causing many environmental problems. Analysis of microplastic pollution in marine water and sediments in Hong Kong has also found ethylene-propylene copolymer among the dominant microplastic pollutants [41]. Nelms et al. [42] discussed the trophic transfer of microplastics in marine to predators (e.g. Grey seals (*Halichoerus grypus*), Atlantic mackerel (*Scomber scombrus*)) and observed ethylene-propylene copolymer is one of the most abundantly found microplastics in top marine predators. A recent study found that 10 commercially caught fish species in the Bay of Bengal, off the coast of Bangladesh recorded an average abundance of 2.2 ± 0.89 microplastics per individual fish. Where ethylene-propylene copolymer contributed 2 % of these microplastic loads [43]. Also, an ethylene-propylene copolymer is observed in edible nori (*Pyropia* spp.) as microplastics tend to attach to marine macroalgae [44]. Furthermore, in terrestrial environments, ethylene-propylene copolymer pollution has been observed. In Shouguang City, Shandong Province, the largest greenhouse vegetable production area in China, ethylene-propylene microplastics were found to be polluting the soil [45].

Product, FW-02 was found to be including Ethyl cellulose (EC) as an exfoliating agent. EC is a linear polysaccharide, cellulose derivative [46,47]. Since EC exhibits hydrophobic properties, Nile red analysis gave positive results even though it is not a microplastic. In cosmetics and personal care products EC is mainly used as a binder, coating material, microspheres and to control the release of active ingredients [48].

The presented data highlights the importance of environmentally friendly, economically feasible, and healthy alternatives for synthetic microplastics or microbeads. One of the most efficient ways of replacing non-degradable plastic microplastics or microbeads as exfoliants is to use natural ingredients such as apricot kernels, jojoba beads, rice bran, almond shells, etc. As Hunt, Lin and Voulvoulis [49] discussed average price per kilogram of nylon, polyethylene, and polypropylene costs 95USD, 50 USD, and 0.90USD respectively, whereas apricot, jojoba, and almond shells cost 11USD, 30.50USD, and 6.55USD respectively. While this evidence supports the financial feasibility of the usage of natural products as exfoliants, more studies must be conducted to find out consumer desirability and potential changes of products that may arise from using the alternatives. The next alternative is to use biodegradable beads. Since the major problem with the usage of synthetic microbeads is their non-degradable nature, many recent studies have been conducted to develop biodegradable microbeads that have the same desirable qualities as synthetic ones. Chitin microbeads made up of waste shrimp shell, Electro sprayed Ca-alginate microbeads, poly(3-hydroxybutyrate-*co*-4-hydroxybutyrate) microbeads and biodegradable polyesters are few of the possible alternatives [[50], [51], [52], [53]]. Apart from these powdered exfoliants (e.g. sugar, salts) and natural clays (e.g. mica) are discussed as viable alternatives.

To understand the absolute nature of microplastic availability in personal care and cosmetic products in Sri Lankan markets, comprehensive studies should be conducted. This research, however, is subject to several limitations. One of the limitations was the number of products tested. Due to fund limitations, the present study only analyzed 15 brands in triplicates. Thus the real situation may not have been demonstrated through this study as there are hundreds of product brands available in the country. Further, as a preliminary microplastic screening step, the present study used Nile Red staining. However, the accuracy of this method is subject to discussion. While many studies successfully utilize this procedure for microplastic analysis, other studies question the accuracy of it. Thus it is better to subject all the isolated particles to the FT-IR analysis irrespective of the Nile Red staining result. Finally, the lack of proper instruments and funds has posed a disadvantage throughout this study. Hence, it is important to overcome these shortcomings during further studies.

## 4 Conclusion

Personal care products and cosmetics are highly utilized products in modern society and in many instances, these products have been acting up as potential health and environmental adversaries. Therefore, the present study discussed 5 categories of personal care and cosmetics products (face wash, facial scrubs, skin creams, baby products, and shaving creams) and have been analyzed for their microplastic composition.

The present study showed that 6 out of 15 products analyzed contained microplastic particles. Ethylene propylene copolymer was the most found polymer followed by low-density polyethylene. In one product ethyl cellulose microspheres were found which demonstrated positive Nile red staining along the other 6 polymers. While ethyl cellulose is not a synthetic polymer it can be assumed that such microspheres were added to increase the exfoliation and to add extra bulk to the product. Emission calculations of microplastics were only possible for two products due to the non-isolatable nature of microplastics found in the other 4 products. Nevertheless, FW-03 contained 0.2 g of low-density polyethylene particles while FS-01 contained 3.36 g of low-density polyethylene particles in the product. Morphologically, these particles were irregularly shaped and white-colored.

Further, this study's results provide a much-needed insight into the personal care and cosmetics products in Sri Lanka. Since microplastics in these products have the potential to cause devastating effects on the environment in the long term, it is essential to provide immediate and impactful solutions and management steps. Therefore, the study results could act as baseline research for future studies and policy development activities. At the end of this study following recommendations could be presented to manage the pollutants and other social aspects related to personal care and cosmetic products.

• Existing regulations should be followed by product developers. If the existing regulations prove to be obsolete, new and improved regulations and state policies must be developed. Regulatory bodies of the state have a significant role to play in this process as they should actively monitor the adherence to policies by manufacturers, rather than being a passive entity.
• Extended producer responsibility policies should be enforced. By doing so cost of the environmental pollution and adverse health effects could be internalized rather than externalized which creates an additional burden on the governments and the general public.
• Producers should be held accountable for designing environmentally friendly packaging and using healthy ingredients. Rather than using synthetic and environmentally unfavorable materials manufacturers should go for sustainable raw materials. To do so new research and development activities should be conducted comprehensively.
• Consumers should be well-informed about products and their consequences.

Extensive and well-organized studies should be conducted regarding the microplastic contamination of consumer products. As this is a growing problem proper attention must be given to tackle this problem.

## Funding

This research did not receive any specific funding from funding agencies in the public, commercial, or not-for-profit sectors.

## Data availability

Data will be made available on request.

## CRediT authorship contribution statement

**Sachith Gamage:** Writing – original draft, Methodology, Investigation, Formal analysis, Data curation, Conceptualization. **Yohan Mahagamage:** Writing – review & editing, Supervision, Project administration, Methodology, Investigation, Data curation, Conceptualization.

## Declaration of competing interest

The authors declare that they have no known competing financial interests or personal relationships that could have appeared to influence the work reported in this paper.
